# Bariatric surgery for obese children and adolescents: a review of the moral challenges

**DOI:** 10.1186/1472-6939-14-18

**Published:** 2013-04-30

**Authors:** Bjørn Hofmann

**Affiliations:** 1 Section for Health, Technology, and Society, University College of Gjøvik, PO Box 191, Gjøvik, N-2802, Norway; 2 University of Oslo, PO Box 1130, Blindern, Oslo, N-0318, Norway

**Keywords:** Bariatric surgery, Children, Adolescents, Ethics, Moral

## Abstract

**Background:**

Bariatric surgery for children and adolescents is becoming widespread. However, the evidence is still scarce and of poor quality, and many of the patients are too young to consent. This poses a series of moral challenges, which have to be addressed both when considering bariatric surgery introduced as a health care service and when deciding for treatment for young individuals. A question based (Socratic) approach is applied to reveal underlying moral issues that can be relevant to an open and transparent decision making process.

**Discussion:**

A wide range of moral issues with bariatric surgery for children and adolescents is identified in the literature. There is a moral imperative to help obese minors avoiding serious health problems, but there is little high quality evidence on safety, outcomes, and cost-effectiveness for bariatric surgery in this group. Lack of maturity and family relations poses a series of challenges with autonomy, informed consent, assent, and assessing the best interest of children and adolescents. Social aspects of obesity, such as medicalization, prejudice, and discrimination, raise problems with justice and trust in health professionals. Conceptual issues, such as definition of obesity and treatment end-points, present moral problems. Hidden interests of patients, parents, professionals, industry, and society need to be revealed.

**Summary:**

Performing bariatric surgery for obese children and adolescents in order to discipline their behavior warrants reflection and caution. More evidence on outcomes is needed to be able to balance benefits and risks, to provide information for a valid consent or assent, and to advise minors and parents.

## Background

Obesity has become a pressing health problem worldwide [1], not only for adults but, also for children and adolescents [2-4]. It has been characterized as an epidemic, a surge and a crisis [2,4-8]. Obesity in children and adolescents is associated with serious health consequences, such as hypertension, dyslipidemia, insulin resistance/diabetes, fatty liver disease, obstructive sleep apnea, and psychosocial complications [4,9].

Even though prevention of obesity in children and adolescents has obtained substantial attention, the evidence on effectiveness of preventive measures is often of poor quality [4,7,10-12]. As the number of obese children and adolescents increases, there is a push for action [2-4]. Several treatment options are available for young obese persons [7,13-19]. Life-style interventions and other non-pharmacological treatments are often considered to be the first option but, such interventions have variable outcomes [10,14,20]. Pharmacological treatment appears to have modest effectiveness when combined with lifestyle interventions but, is associated with more adverse effects than lifestyle interventions alone [20,21]. Several surgical procedures are available for children and adolescents but, long term effects from high quality studies are scarce [19,20].

The enthusiasm and apparent success of bariatric surgery in adults has kindled an interesting debate on performing bariatric surgery in children and adolescents. Bariatric surgery for children as young as 5 years old has been reported [22]. However, children and adolescents are still developing, both physically and mentally [14,23-25], they may have reduced competency to consent [26,27], and bariatric surgery may change their life in a substantial way.

The pressing question in this debate is whether we should normalize bariatric surgery for children and adolescents. There is an imperative to help obese minors to avoid serious health problems in the best possible way, but the lack of high quality evidence on safety, outcomes, and cost-effectiveness for bariatric surgery in this patient group makes it difficult to do so. Moreover, lack of maturity and strong family relationships pose a series of challenges related to autonomy and informed consent/assent. Social aspects of obesity, such as medicalization, prejudice, and discrimination, raise problems with justice and trust in health professionals. Conceptual issues, such as the definition of obesity and treatment end-points, are morally demanding along with the handling and balancing of interests of patients, parents, professionals, industry, and society. These, and many other moral challenges, will be presented and discussed in this article. They are important in order to make open and transparent decisions on bariatric surgery for children and adolescents.

Bariatric surgery refers to a variety of surgical procedures, including gastric bypass, adjustable gastric banding, sleeve gastrectomy, and duodenal switch, and includes both open and laparoscopic procedures [25,26]. Due to restricted space (and limited clinical evidence), this article will not analyze each surgical procedure in detail, but discuss moral issues that are common to the various procedures in bariatric surgery which, call for attention. Moreover, there are significant differences between young children and old adolescents and there is substantial variability in maturity within the same age group. Nevertheless, many moral questions are common. When there are specific differences between children and adolescents or special age groups are addressed, this will be made explicit in the text.

This article presents an overview of the many moral challenges that exist with bariatric surgery for children and adolescents. Corresponding reviews for obese adults already exist [28-30] but, children and adolescents are special and deserve distinct attention.

## Methods

To identify the moral issues at stake with bariatric surgery for children and adolescents, a question based (Socratic) approach was applied. A series of morally relevant questions are posed to highlight overt and covert value issues with regard to a medical intervention. The approach is described in detail elsewhere [31-33] and is implemented in a core model of Health Technology Assessment [34-36]. It is used for a wide range of health technologies, including surgery [28,29,37,38].

The aim of the approach is to highlight the moral issues in an open and transparent manner by revealing underlying conceptions and hidden presumptions. It does not analyze bariatric surgery for children and adolescents within a particular ethical framework and does not provide specific recommendations. The objective is to inform decision makers (on various levels) about values, viewpoints, and arguments which appear to be important for actual decisions in context.

### The literature search

MEDLINE, EMBASE, PsycINFO, Web of Science, Eurethics, Bioethics Research Library at Georgetown University, BELIT, and SIBIL was searched for systematic reviews, primary studies, reports and books. Search words were: obesity, obese, overweight, child*, adolescen*, pediatric*, young*, teen*, youth*, ethic*, moral*, patient autonomy, consent, assent, conflict, interest, self determination, health disparities, discrimination, mental capacity, mental competency, parental, perceptive discrimination, and beneficence. The search was performed in October 2012. Titles and abstracts were screened for morally relevant issues. Selected references were assessed for content and clarity of presentation. Articles only mentioning that there are moral challenges, but without explaining or analyzing them, were excluded. Relevant references found in the reviewed literature, were added.

## Results

**Figure 1:**
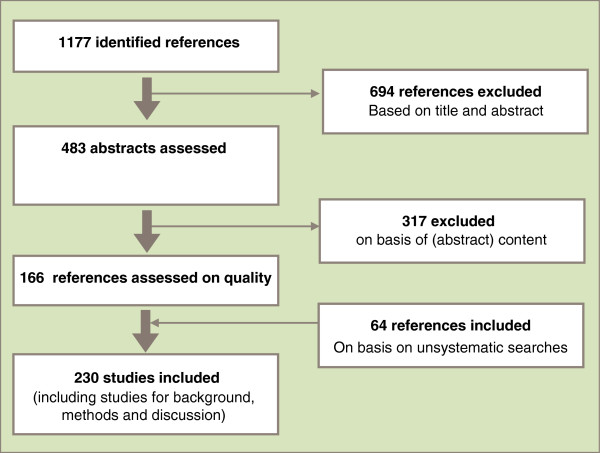
Results from literature search.

The literature search resulted in 1177 references which were processed according to Figure 1.

How the morally relevant questions are addressed, and the related arguments, are presented thematically in the following sections.

### Beneficence and bariatric surgery

Does bariatric surgery benefit children and adolescents? What are the long term outcomes? What is the efficacy, effectiveness, and efficiency of bariatric surgery? Utilitarian assessments of bariatric surgery for young people are difficult as high quality knowledge on benefits is scarce[39]. Knowledge from uncontrolled series’ from selected centers indicates that bariatric surgery may be beneficial [40], but, evidence on important outcome measures is lacking [19,25]. A systematic review and meta-analysis found clinically significant BMI reductions for both Laparoscopic adjustable gastric banding (LAGB) and Roux-en-Y type gastric bypass (RYGB) [41]. Surgery was reported to resolve some medical conditions including diabetes and hypertension but, this was poorly reported. Band slippage and micronutrient deficiency were the most frequently reported complications for LAGB, but band erosion, port/tube dysfunction, hiatal hernia, wound infection, and pouch dilation were also reported. More severe complications have been documented for RYGB, such as pulmonary embolism, shock, intestinal obstruction, postoperative bleeding, staple line leak, and severe malnutrition [41]. A more recent level 1 evidence study shows greater weight loss for those who underwent laparoscopic banding procedures than for those following a medical weight loss program [42]. However, the latter group showed substantial improvement in hypertension, hyperlipidemia, and insulin resistance. New non-invasive procedures trying to mimic hormonal effects of bariatric surgery may become available [43], but, need testing before proper assessment.

History has shown that extrapolating results from adults may be flawed. More evidence is needed. Moreover, the conditions under which bariatric surgery is performed appears to be highly relevant for the outcome, such as preoperative evaluation by a multidisciplinary team, bariatric surgery competency with children and adolescents, postoperative care and follow up [4], and family support [25]. The same goes for timing, operations may be performed too early (with a poor benefit/harm ratio) and too late (missing relevant health benefits) [44,45]. This makes it difficult to balance outcomes against risks [46,47], and to plan post-procedural follow up both on an individual (micro), institutional (meso), and a societal (macro) level. Moreover, with bariatric surgery for children and adolescents the question of “benefit for whom?” becomes pertinent, as will be discussed in further detail below.

### Safety and risk

The risks and complications of obesity-related comorbidities are definite and life-threatening [4]. This may justify fervent interventions. However, bariatric surgery is associated with serious risks and recognized complications, which appear to be relevant [41]. As stated by Han and co-workers, “The risks of bariatric surgery are considerable, and its long-term safety and efficacy in children remains largely unknown. Therefore, surgery should be reserved for only the most severely obese (BMI ≥ 50 kg/m2 or BMI ≥ 40 kg/m2 with significant co-morbidities), and even then, considered with extreme caution” [20]. Moreover, adverse events are reported to be frequent even with very experienced surgeons [12,48].

Most published studies measure anthropometric and metabolic outcomes but, psychosocial effects may be relevant. Childhood and adolescence is characterized by intense psychological and social development and turbulence. Only a few studies have investigated psychosocial effects of bariatric surgery and the results are short term and based on small patient series’ [49]. Some studies indicate significant rates of high depression scores and negative self-acceptance in adolescents after laparoscopic adjustable gastric banding (LAGB) [50]. When bariatric surgery does not work as expected, people can feel shame and guilt [51], further enhancing the burden of being obese. Moreover, persons evaluated for bariatric surgery showed clinically significant depressive symptoms [52]. This indicates that there is a need for psychological monitoring before and after bariatric surgery and for additional psychosocial support to be available for vulnerable sub-groups of adolescents [49,53]. A significant rate of unplanned pregnancy within the first 2 years after bariatric surgery has been reported in female adolescents [54].

Due to the learning curve of bariatric procedures, low complication rates are typically seen after 100–150 procedures which, indicates that bariatric surgery in children and adolescents should be performed in specialized, high volume centers [25]. Accordingly, assessment of safety and risks may be complex and challenging.

### Autonomy and compliance

The reduced autonomy and vulnerability of children and adolescents pose challenges to decision making [55]. Bariatric surgery will influence a person’s everyday life and restrict their life-style choices. This raises the question of whether surgery decreases or increases patient autonomy. Obesity is frequently characterized as a life-style disease and eating is considered to be an individual’s act of free choice. Obesity is therefore sometimes conceived of as a weakness of character [56,57] or a weakness of the will (*akrasia*). At the same time, it is argued that obesity may have a genetic origin, reducing the person’s autonomy. In both cases, it can be argued that the need for bariatric surgery results from lack of autonomy. However, a potential lack of control with eating does not necessarily reduce a person’s ability to assent to bariatric surgery or other health care issues. Accordingly, obesity is not a general sign of reduced autonomy. Quite opposite, as weight reduction can increase a sense of control and self-esteem, bariatric surgery may increase autonomy, e.g. some feel that they gain control because treatment limits their choice and imposes control over their eating habits [58]. However, the evidence for such effects is limited.

Health interventions aimed at obesity may be in the best interest of the common good, as obesity in children and adolescents is expected to become a significant health problem. However, such interventions may become paternalistic and infringe on personal autonomy [59]. There are many other values involved than health [60] and there are limits to intrusion on basis of a common good, even in the area of health [59,61,62].

As children and adolescents may have reduced autonomy, they will assent. Parents will frequently give consent for operations for their minor children [63]. However, parents may have different conceptions of the seriousness of obesity and diverging interests [64]. It is generally assumed that parents are in the best position to know what is best for their children but, for some obese children this presumption can be questioned [65] (see below). Assessing what is in the best interest of the child is demanding, especially in cases where there is justified doubt whether the parent(s) are able to manage their child’s autonomy.

Furthermore, obesity treatment can be interpreted as a way of disciplining human bodies. Exercise and dietary therapy may be seen mainly as an external disciplining of the human body through the regulation of behavior. Accordingly, pharmacological therapy is a disciplining of human behavior by medical intervention, and bariatric surgery is disciplining through organ modification. If such disciplining hampers or alters voluntariness, personal autonomy is challenged.

The intervention hierarchy often demands life-style interventions (and pharmacological interventions) before surgery [66]. This can be seen as a restriction of choice and autonomy, and its effectiveness and efficiency is not well documented [67], especially in children and adolescents. However, the intervention hierarchy may find its justification in the precautionary principle more than from evidence on effectiveness and efficiency. Patient autonomy, (i.e., of children and their parents) may also be curbed by professionals’ strong opinions about treatment options (discussed below).

It is also argued that patients “should be able to demonstrate adherence to a lifestyle modification program prior to surgery and the ability to comprehend and be able to cope with nutritional and behavioral ramifications of bariatric procedures” [25]. Here, reduced autonomy is linked to expected results of bariatric surgery. Lack of compliance is believed to give a poor outcome. Some procedures, such as laparoscopic banding, appear to require more patient compliance than e.g. RYGB [12], and concerns exist whether young people are able to comply with rigorous follow up. However, paternalism may also be lurking, “An adolescent who is not reliable in other aspects of his/her life, ‘forgets’ school assignments or household chores, and frequently misses appointments may not be a suitable bariatric candidate.” [63]. It may be erroneous to infer from the autonomy in one area of life to the autonomy in another.

### Informed consent

#### Information disclosure and understanding

It is difficult to inform eligible persons in a way that ensures valid informed consent [68,69] or assent as the evidence of long-term effectiveness and safety of bariatric procedures is not clear. It is especially challenging to inform individuals about potential harms and uncertain long term benefits and to inform young people about prospects that they find hard to identify and recognize. Age, maturation and psychological state are important aspects for assessment [70,71]. Studies show that patients undergoing bariatric surgery do not remember information on potential complications [72,73]. Furthermore, the quality of information on bariatric treatment on the internet provided by medical centers and professional organizations is variable [74]. Children and adolescents with severe obesity may also have difficulties with understanding and assessing information due to depressive symptoms [52].

Patients, parents, and surgeons may be overly optimistic [63]. Provided information can be biased or poorly prepared for children and information may be received and read with strong preferences and preconceptions. Relevant information may not be disclosed such as, variability in treatment outcomes from various health care providers [63], how bariatric treatment may change the daily life of the person in significant ways, and the sometimes close relationships between health care professionals and industry [75], e.g. with regards to drugs, devices and surgical banding techniques. Moreover, health illiteracy is a challenge [69], especially in children and adolescents. Patients and parents may also seek surgery because they are desperate or have become scared [63]. Hence, informing children and adolescents is even more challenging than informing adults eligible for bariatric treatments in order to obtain valid informed consent [27,76].

#### Competence to consent or assent

As already indicated, obesity is associated with psychiatric comorbidity [77-86], reduced cognitive abilities [87,88], and verbal skills [89], affecting patient autonomy and competence to consent [90]. Obesity is related to anxiety, depression, and impaired self-esteem [91-95] as well as impaired social relationships [96]. About one third of the adult candidates for bariatric surgery were identified as victims of sexual abuse as children [6,97-99], and many were subject to childhood mistreatment [97]. Past abuse appears to be overlooked during assessment for bariatric surgery [100]. It is argued that assessing mental capacity in young surgical candidates is important, and psychiatric comorbidity may be relevant [66] and should be screened for [101].

#### Voluntariness

Children, adolescents and parents may have different conceptions of obesity [102] and of its impact on their lives [103-107]. Parents tend to focus on the negative medical and psychosocial impact of obesity. Thus, there is a risk of (overt or covert) coercion in the child or adolescent’s assent or consent [16,63]. It is argued that it is important to assess the cognitive, emotional, and social development as well as support for the child and adolescent’s independence [108]. Caniano provides a useful checklist for assuring valid consent for surgery on children and adolescents and underscores the importance of making sure that patients and parents understand “the irreversibility and likelihood of unanticipated negative consequences several years later” [63].

Minors’ rights to consent to surgery are regulated differently in different countries. In many countries minors can give consent to surgery from the age of 16. Parents may consent for children but, for serious interventions, children may have to assent (i.e., they have to be informed in a way that they understand, and solicit their reactions). This intensifies the above mentioned challenges with informed consent. There are also other examples where bariatric surgery for children and adolescents comes up against existing laws and regulations in various countries [61,62]. However, the legal aspects are beyond the scope of this article.

### Prejudices

Obese persons are subject to several kinds of prejudice because of their weight [109,110]. Weight discrimination and stigmatization is well documented and appears to be rapidly increasing in adults [93,111-115] as well as children [116-119]. Typically this takes the form of disparities in access to health-care facilities [120] and to education [57]. Negative stereotypes exist, e.g., that overweight and obese persons are lazy, sloppy, unmotivated, noncompliant, less competent, “willful deviants”, and that they lack self-discipline [56,57,121-125]. Such stereotypes also exist among health professionals [104,125-129]. Obese persons are subject to “invalid stereotypes”, “fatism”, and “weight-harassment”, and should be regarded as victims [130]. Although terms like “obesity epidemic” and “obesity gene” highlight environmental or genetic factors and indicate that obesity is something which happens to people more than a result of their choices [131-133], obesity is frequently considered to be a personal responsibility [134,135]. Although this responsibility may be projected on parents, children still feel guilt and shame for their obesity (see below).

Health professionals tend to be pessimistic about obese people’s ability to manage their situation [136], and are sometimes reluctant to refer children to bariatric surgery [137,138]. Almost half of the family physicians and pediatricians participating in an American study stated that they would never refer an obese adolescent for bariatric surgery [138]. One reason for this appears to be the scarce high quality evidence and fear for complications.

Stereotypes and prejudices result in discrimination of appearance and threaten the integrity and dignity of young people with high body weight who are in a vulnerable situation and a susceptible phase of development. This also raises the question of whether pharmacological and surgical interventions are medical solutions to social problems (of unsound attitudes and discrimination) [28,29].

### The social phenomenon of eating and the social construction of obesity

Food and eating have strong social and cultural bearings. At the same time they are considered to be profoundly private [139]. Bariatric surgery changes people’s eating habits and preferences, routines, medication regimens, and ability to socialize. Accordingly, bariatric surgery may represent an extensive intervention in people’s daily living. On the one hand, this may not seem important to children and adolescents at the time of intervention, however, it may become important to them later in life. On the other hand, improvement in body weight and increased control with food intake, as well as altered habits, may have positive effects on social behavior. Surgery may enforce a structured lifestyle.

As obesity is associated with somatic diseases, body ideals, and lack of self-control [59,60], it may be argued that bariatric surgery consolidates existing social condemnation of fat [5,59] and sustains existing aesthetic ideals of body and beauty [140]. Like other diseases, such as HIV, the meaning of (severe) obesity goes beyond its actual pathology, e.g., as an indicator for social status or moral weakness [131]. Interventions towards obesity have complex social effects, such as social anxieties about fat and physical appearance [141], and are difficult to gauge [142]. Moreover, defining obesity for children is more difficult than for adults, in part because the evidence on morbidity and mortality for obesity in children are not as prevalent as in adults, as obesity complications frequently take years to develop [143,144].

There are many social perspectives on “the obesity epidemic” in the literature which, have important moral implications [145,146]. The obesity epidemic can be interpreted as a social construction by government health agencies, academic obesity scientists and researchers, obesity associations, health care professionals with the aim of creating a market, and industry [131,147]. It has also been interpreted as a moral panic driven by an ideology of gender, class and race [148-156], and as a tool for the politicization of body size, i.e., making private issues subject to public regulation [131,157]. It is argued that the construction of the "obesity epidemic" has less to do with health consequences of excessive weight than with various financial and political incentives of the public health bureaucracy, weight loss industry, and the medical profession [131,150]. Obesity has also been interpreted in terms of the food industry and regulations [158-163]. Bariatric treatment may be viewed as part of medicalization of people’s private sphere and daily life [30,131,164]. Accordingly, bariatric surgery is a way to correct unwanted moral behavior (calorie intake) transforming persons into patients [5], and maintaining the “health industry” [131]. Such power perspectives are even clearer with children and adolescents.

The point here is not that these perspectives are true but, that they point to important moral and social issues. As the etiology of obesity is complex and still unclear, bariatric surgery can be conceived of in many ways and some of these may be oppressive and discriminating, especially for children.

### Equal access and justice

Bariatric surgery is costly and may drain resources from other parts of health care. At the same time it may avoid significant future health costs. Overweight and obesity is unevenly distributed amongst children and adolescents [165-167], and those of lower socioeconomic status and minority status are disproportionately affected [168-173]. There appear to be differences between ethnic groups [174-177]. In the USA obesity affects one in three socially disadvantaged children. African American girls and Hispanic and Native American children of both genders have high rates [63,178]. Hence, it is likely that there is (and will be) an uneven access to bariatric surgery in children and adolescents in the same manner as there is in adults [99,179]. A difference in access challenges norms for equality [180]. However, as the evidence for the safety, effectiveness, and efficiency for bariatric surgery for children (and adolescents) is not yet convincing, it is difficult to assess its distributive justice. Besides, there may be many reasons for unequal distribution of bariatric surgery. The criteria for selection of eligible and motivated candidates for pediatric bariatric surgery is but one of these [27,52,105].

### Stakeholders’ interests

Health professionals are stakeholders with professional and economic interests [181]. Their main interest is to help in what they conceive of to be the best way, but they may also profit from “the obesity epidemic” [5,131,182]. Surgeons may have invested time and prestige in particular procedures and may have close relationships with industry. Some surgical procedures require frequent follow ups and ensure activity. Industry has reasonable interests in selling equipment and devices. Society may have significant long term interests in helping young people to avoid developing serious and costly diseases. Moreover, information providers may profit from communicating success stories about young people becoming slim and happy after bariatric surgery.

Parents are mostly devoted to the best interest of their children but, this does not exclude some of them from seeing their child’s obesity as a personal failure and that they find surgery as a convenient solution. Moreover, the best interest of the child is difficult to assess. Children undergoing surgery may, later in life, come to regret the decision (of their parents) and the intervention by health professionals. This points to a responsibility both for parents and professionals.

### Responsibility

Bariatric surgery for children and adolescents differs from surgery for adults in that a third party is involved. Parents are responsible for the upbringing of their child, including their nourishment [183]. Children have the right to a healthy environment [184,185]. It is argued that a children’s rights perspective can propel more effective health interventions [186]; that parents who do not listen to advice should be targeted under child protection laws [187], that children should be removed from their home under certain conditions [65], and that state intervention is justified in extreme cases [188-191]. As some parents are (partly) responsible for their child’s obesity [192-195], surgery can be viewed as a quick fix for neglected parental responsibility. Childhood obesity may justify enforced treatment outside the home in the case of neglect [65]. Parental responsibility may also be relevant in assessing children (and families) eligibility for surgery, as parents’ involvement is important in pre-adolescent and adolescent treatment [196].

In contrast, parents may feel guilt for their child’s situation, even when such feelings are not justified. It is argued that parental responsibility should have no influence on access to surgery [197], that although parents are causally and morally responsible, they may not be blameworthy [198], and that interventions in the families are not justified [199]. It is also argued that personal and parental responsibility should be analyzed in a social perspective as a matter of public health [178,200-202], and that creating healthy defaults can bridge the divide between individual and social responsibility [160,203-205].

### Lack of knowledge – a moral imperative

The present lack of high quality evidence on outcome and risk in bariatric surgery for children and adolescents is a moral quandary [181,206,207], and voices a moral imperative to provide more high quality knowledge. This of course presupposes that it is acceptable for children and/or adolescents to participate in research with bariatric surgery. Despite some reluctance in the professional community, it is argued that as long as high quality evidence of long term outcome is lacking, bariatric surgery for children and adolescents should be considered innovative treatment [63] and that “surgery should be performed in institutions that are equipped to meet the tertiary care needs of severely obese patients and to collect long term data on the clinical outcomes of these patients” [14]. Children and adolescents enrolled in studies should be treated at centers with special expertise in care of this group [208]. Therefore, there is an imperative to do proper research [63], but, research on children and adolescents poses a series of challenges, especially assessment of risks and benefits and informed consent [209,210]. Research on vulnerable groups entails special caution [211,212], especially as scientists may have paternalistic attitudes and prejudice [213].

### Conceptual challenges

So called factual issues, such as obesity prevalence and treatment outcome, have normative (moral) underpinnings. They depend on the definition of obesity, which has changed over time [18,214,215], and is still not clear, especially not for children [216]. The selection of classifications of nutritional status in children and adolescents, references, cut-offs, and terms to be used in different contexts are issues with moral foundations and implications, e.g., who should be treated and how we assess success.

While there is some international consensus on thresholds of body-mass index (BMI) for defining overweight and obesity in adults, the effects of age, gender, pubertal status and race/ethnicity on growth make classification difficult for minors [20]. Consensus on definition and classification is of vital importance to obtain high quality knowledge [217].

Obesity is not a category in nature, i.e., it is not a natural kind [218], but rather a social construction established to find ways to help people considered to be vulnerable and in need of aid. It is unclear whether obesity, in itself, is a (biomedical) disease, and whose problem it is [58,219-222]. The WHO considers obesity to be a disease, but not all medical experts agree [56,131]. Members of The Obesity Society (TOS) point out that obesity is not a disease in a strict scientific sense but, a normative concept suitable to obtain professional attention and respect [223]. Even where obesity is accepted as a disease, there is little agreement on what kind of disease it is, e.g. whether it is a psychological, physiological, metabolic, esthetical, or behavioral disease. Moreover, widespread use of surgery can alter obesity from being a typically “medical disease” to become a “surgical disease”.

Trying to differentiate morbid from non-morbid obesity leads us back to square one; the challenge of defining cases of obesity eligible for treatment. Focusing on underlying biological causal factors is so far of little help, as their interaction is complex and unclear. Moreover, the underlying biological phenomena we pay attention to may be addressed because we think they deserve medical attention, resulting in a circular argumentation. Furthermore, to alter a normal and well-functioning organ in order to discipline human behavior may appear conceptually and morally challenging [224]. Hence, conceptual questions, such as whether obesity is a disease and how it is to be defined, pose a series of moral issues with bariatric surgery, especially for children and adolescents.

## Discussion

Some studies indicate that bariatric surgery may be effective on short and medium term weight loss which, is associated with a reduction of comorbidities, such as diabetes. Furthermore, it is generally believed that several types of bariatric surgery are cost-effective. This poses a moral imperative to provide surgery to a vulnerable group of patients in need of help. However, high quality evidence on safety, efficacy, effectiveness, efficiency, and cost savings for children and adolescents is lacking. Exposing young people to potentially harmful treatment with uncertain outcomes is morally problematic. Hence, it is difficult to assess the risk/benefit ratio [63] and there is a moral imperative to provide high quality evidence.

Moreover, bariatric surgery for children and adolescents poses predicaments with informed consent and assent due to the complexity and uncertainty of information, reduced decision making capacity (e.g., due to potential psychiatric comorbidity), and lack of voluntariness due to family bonds. As obesity is frequently considered to be a life-style disease, it is associated with parental responsibility. The involvement of third party agents (parents) and the association with responsibility and guilt complicate issues of consent, assent, justice [30], and the assessment of the best interest of the child.

Obesity is subject to prejudice and discrimination, posing challenges with prioritization and just distribution of health care. As prejudice is identified also among health professionals it may alter the patient-professional relationship and trust in the health care system. Widespread bariatric surgery for minors can advance ideals on health and beauty which, may be part of the primary problem. Hence, bariatric surgery is more than a mere medical intervention shaping biological bodies – it shapes and is shaped by culture.

Whether obesity is a disease, how it is defined and classified, and the selection of end-points for outcome assessment strongly depends on social commitments and moral conceptions, e.g., on what we believe to be harmful and how we think we can provide the best help. Accordingly, it may be important to avoid the critique that bariatric surgery for obesity in minors is a medicalization of their life-world and a quick fix [225].

The ethical analysis in this review does not end with simple answers or concrete recommendations. The reason for this is fourfold. First, the (Socratic) approach does not aim at presenting clear cut conclusions. Rather it aims at revealing important moral issues that are relevant for open and transparent decision making processes. Second, decisions have to be made in context and contexts are different, e.g., the conceptions of the autonomy of minors is assessed differently in different countries. Arguments have to be assessed, values weighted, and alternatives appraised in the context of decision making. Third, the conclusions may change rapidly as new methods and new evidence emerges. Still, many of the moral issues are generic and may be relevant and have to be addressed in order to implement and offer bariatric surgery to obese minors in a morally acceptable way. Fourth, this review is written by an ethicist with special interest in surgery, biotechnology, and health technology assessment. Despite this competence, it is far from obvious that the opinion of experts in ethics should have special weight or priority [226-228]. Others may be as well qualified. In particular, we should listen to the group in question, and their voice is not always loud or apparent in the professional literature.

The analysis presented here displays the values at play in, and related to, the assessment of bariatric surgery for obese minors. In particular, it tries to highlight the evaluative aspects of what is regarded as fact. In that manner it does the preparatory work for the decision making process. Even more, it tries to direct the process towards openness and transparency. Ignoring unpleasant, but important, value issues may become more difficult when they have been explicitly pointed to and highlighted.

Moreover, the selection of questions and challenges discussed in this review is by no means value neutral [31,32]. Nevertheless, the review does not represent specific interests, such as patient interest groups, surgeons, industry, health care managers, health insurers, or health policy makers.

Other methods may, of course, have been applied [35,36]. Nevertheless, the approach applied here is fairly well established for assessing health technologies and is able to highlight many of the challenges that are identified in the literature. It has also been applied to bariatric surgery for adults [28] and bariatric treatment for adults [29].

Sources of data other than the professional and scientific literature could also have been applied. In particular, primary studies with qualitative interviews of eligible persons for bariatric treatment, surgeons, industry, health insurers, and health policy makers could shed new light on the issue. However, primary research has been beyond the scope of this review.

How specific are the reviewed moral challenges for bariatric surgery? Are they as relevant for other health care interventions as well? This may well be, but bariatric surgery poses particular quandaries for minors because it uses medical interventions to alter everyday behavior (diet therapy, exercise, cognitive-behavioral therapies), as well as modifying organs and processes that otherwise appear healthy and because it does not remove the multifarious complex and in part unknown causes of obesity on persons who often cannot give valid informed consent or assent. It provides no cure but, offers symptom relief and prevents other diseases. Moreover, the disease that bariatric surgery is directed at alleviating is special in that it is considered to be self-inflicted, resulting from lack of self-control, and is subject to prejudice. The evidence for the outcome is also of poor quality for minors.

Accordingly, it can be argued that to operate or not to operate, is not the question. The important questions are; when is the right time for surgery? Which are the right patients? How should they be prepared for surgery? [25], and how should they be followed up? [25]. Surgery very early in the development of obesity can have serious consequences, but surgery late in the development of severe obesity may also be harmful [99,229]: ‘[a] stitch in time versus a life in misery’ [230]. This dilemma corresponds well with a long tradition in medical ethics framed by the Greek concept *kairos*, finding the right time for intervention. It also reminds us of Macbeth: “one must ‘make assurance doubly sure.”

## Summary

In sum, there is not one answer to the question of whether bariatric surgery should be performed on obese children and adolescents. To some minors, bariatric surgery may be the only option in order to save their lives or to avoid severe disease. For others, bariatric surgery may be morally wrong, e.g. if more beneficent alternatives exist. This review has intended to highlight a broad spectrum of moral issues that have to be addressed to make decisions on whether to perform bariatric surgery on children and adolescents. In particular, decision making under uncertainty, informed consent or assent, prejudice, discrimination, and justice have been highlighted. These issues are relevant for decisions on the micro, meso and macro level, and they have to be addressed in context in order to make sound, open and transparent decisions on bariatric surgery for children and adolescents. Moreover, the moral issues highlighted in this review may be crucial in providing a morally justified and sound health service. Therefore, to cut or not to cut, is not the question. The important questions are; who to operate on, when to do it, who is to decide, how to decide, who is to operate, how best to prepare, and how to follow up, and last, but not least, how to generate more high quality evidence in a morally acceptable manner. Additionally, we should ask ourselves how to avoid prejudice and discrimination as well as surgery due to body ideals or parent’s feeling of guilt and failure.

Cutting into children’s healthy organs in order to discipline their behavior, to satisfy social ideals for body shape, or to compensate for poor parenting should be avoided. More evidence on outcomes is needed to balance benefits and risks, to provide information for a valid consent or assent, and to advise minors and parents.

## Competing interests

The author has a part time position at the Norwegian Knowledge Centre for the Health Services in Norway, engaged in making Health Technology Assessments on bariatric surgery, but he is not involved in treatment of obese children has no competing interests to declare.

## Authors’ contributions

BH has carried out the study conception and design. Malene Gundersen has assisted in making the literature search. BH has made the acquisition and analysis of data, the drafting and revision of the manuscript, as well as the approval of the final manuscript.

## Pre-publication history

The pre-publication history for this paper can be accessed here:

http://www.biomedcentral.com/1472-6939/14/18/prepub
